# Non-Uniform Survival Rate of Heterodimerization Links in the Evolution of the Yeast Protein-Protein Interaction Network

**DOI:** 10.1371/journal.pone.0001667

**Published:** 2008-02-27

**Authors:** Takeshi Hase, Yoshihito Niimura, Tsuguchika Kaminuma, Hiroshi Tanaka

**Affiliations:** 1 Department of Bioinformatics, Medical Research Institute, Tokyo Medical and Dental University, Tokyo, Japan; 2 Department of Bioinformatics, School of Biomedical Science, Tokyo Medical and Dental University, Tokyo, Japan; Indiana University, United States of America

## Abstract

Protein-protein interaction networks (PINs) are scale-free networks with a small-world property. In a small-world network, the average cluster coefficient (<*C*>) is much higher than in a random network, but the average shortest path length (<*L*>) is similar between the two networks. To understand the evolutionary mechanisms shaping the structure of PINs, simulation studies using various network growth models have been performed. It has been reported that the heterodimerization (HD) model, in which a new link is added between duplicated nodes with a uniform probability, could reproduce scale-freeness and a high <*C*>. In this paper, however, we show that the HD model is unsatisfactory, because (i) to reproduce the high <*C*> in the yeast PIN, a much larger number (*n*_HI_) of HD links (links between duplicated nodes) are required than the estimated number of *n*_HI_ in the yeast PIN and (ii) the spatial distribution of triangles in the yeast PIN is highly skewed but the HD model cannot reproduce the skewed distribution. To resolve these discrepancies, we here propose a new model named the non-uniform heterodimerization (NHD) model. In this model, an HD link is preferentially attached between duplicated nodes when they share many common neighbors. Simulation studies demonstrated that the NHD model can successfully reproduce the high <*C*>, the low *n*_HI_, and the skewed distribution of triangles in the yeast PIN. These results suggest that the survival rate of HD links is not uniform in the evolution of PINs, and that an HD link between high-degree nodes tends to be evolutionarily conservative. The non-uniform survival rate of HD links can be explained by assuming a low mutation rate for a high-degree node, and thus this model appears to be biologically plausible.

## Introduction

The information of protein-protein interaction networks (PINs) at the whole-genome level is now available from several organisms, including *Saccharomyces cerevisiae* [1]–[3], *Caenorhabditis elegans* [4], and *Drosophila melanogaster* [5]. These data were provided by using high-throughput experimental techniques such as yeast two-hybrid screens [1], [2]. The structure of PINs is represented as nodes (proteins) and links (interactions between proteins). Studies of PIN structures have revealed that PINs exhibit the following interesting properties [6].

First, PINs are scale-free networks [7], [8]. The number of links connected to a node is called a degree. The degree distribution *P*(*k*) gives the probability that a node has *k* links (*i.e.*, degree *k*). In a scale-free network, *P*(*k*) decays as a power law, following *P*(*k*)∼*k^−γ^* [9]. (In the case of PINs, it is known that *P*(*k*) better fits a power law with an exponential cut-off, *i.e.*, *P*(*k*)∼(*k*_0_+*k*)^−*γ*^e^−*k*/*k*c^ [7], [10].) Therefore, a scale-free network is highly heterogeneous and is characterized by the presence of a large number of nodes having only a few links and a small number of nodes (hubs) that have numerous links. A scale-free network is known to be tolerant to random removal of nodes, but it is very fragile against selective removal of hubs [7], [11]. Second, PINs are small-world networks [4], [5], [8], [10]. A small-world network is highly clustered like regular lattices, but it has small path lengths like a random network [12]. A small-world property is quantified by two statistics of a network, the average cluster coefficient <*C*> and the average shortest path length <*L>*. The cluster coefficient of node *i* is defined as *C_i = _2e_i_*/*k_i_(k_i_−1)*, where *k_i_* is the degree of node *i* and *e_i_* is the number of links connecting *k_i_* neighbors of node *i* to one another [12]. (When *k_i_* is zero or one, *C_i_* is defined to be zero.) In other words, *e_i_* is the number of triangles that pass through node *i*. *C_i_* is equal to one when all neighbors of node *i* are fully connected to one another, while *C_i_* is zero when none of the neighbors are connected to one another. A small-world network is characterized by a <*C*> that is larger, and an <*L*> that is similar, to those of a random network [12]. (In a random network, <*C*> = <*k*>/*N* and <*L*>∼log*N*/log<*k*> [13], where <*k*> is the average degree and *N* is the number of nodes.) Scale-free and small-world properties are commonly observed in various complex networks such as the Internet [9], coauthorship of scientific papers [14], metabolic pathways [15], and functional connections in the human brain [16]. Third, PINs show a hierarchical structure. In a network showing a hierarchical structure, <*C*(*k*)>, the average cluster coefficient of *k*-degree nodes, decays as a power law <*C*(*k*)*>*∼*k^−μ^* [17], [18]. This indicates that a node with a small number of links has a high *C* and belongs to a small subnetwork in which all nodes are densely connected, while a hub has a low *C* and links different subnetworks. Fourth, PINs show a disassortative structure, in which <*K*_nn_(*k*)> (“nn” represents “nearest neighbors”), the average degree among the neighbors of all *k*-degree nodes, follows <*K*_nn_(*k*)*>*∼*k^−ν^* [19]–[21]. Therefore, the connections between a hub and a low-degree node are favored, while those between hubs and those between low-degree nodes are suppressed [19]–[22].

It has been reported that the emergence of scale-free networks can be explained by the mechanisms of network growth and preferential attachment, in which a new node is preferentially attached to a node that already has many links [23]. In the PIN evolution, gene duplication is thought to be responsible for preferential attachment, because gene duplication creates a new node having the same interacting patterns as the original node, and a high-degree node is more likely to gain a new link by the duplication of a randomly selected node than a low-degree node [6]. To account for the properties of PINs mentioned above, several network growth models have been proposed. These models are generally based on gene duplication and divergence. In a divergence process, some of the links created by duplication are removed and some new links are added to a network. Sole et al. [10] proposed a model in which a divergence process includes two mechanisms, random removal of links from one of the duplicated nodes and random attachment of new links between a duplicated node and another node. Both simulation and analytical studies have shown that this model can generate scale-free and small-world properties [10], [24]–[27]. However, studies have reported that a network generated by this model having the same number of nodes and links as those in the yeast and fly PINs showed a much smaller <*C*> than these PINs [10], [28], [29]. To overcome this difficulty, Vazquez et al. [30] and Ispolatov et al. [29] proposed the heterodimerization (HD) model. In this model, gene duplication is followed by divergence and HD; in the divergence process links are removed from duplicated nodes with a uniform probability *α*, and in the HD process a new link is established between two duplicated nodes with another probability *β*, forming a heterodimer [29], [30]. When a self-interacting protein is duplicated, the duplicated proteins will interact to each other. Therefore, *β* in the HD model represents the probability that a randomly selected protein is self-interacting and the link between two duplicated proteins survives after divergence. Simulation and analytical studies have shown that the HD model could reproduce a similar <*C*> to the yeast and fly PINs as well as a scale-free property [28]–[30]. The reason for the successful reproduction of a large <*C*> is that an HD process creates a triangle, and a network containing a large number of triangles shows a large <*C*>. Middendorf et al. [28] reported that the HD model could best reproduce the fly PIN among seven network growth models using a technique from machine learning.

In this paper, we examine the yeast PIN, since it constitutes the most reliable PIN data currently available at the whole genome level [31]. We first show that the HD model is unsatisfactory as an evolutionary model of the yeast PIN. We then propose a new model named the non-uniform heterodimerization (NHD) model, in which an HD link is preferentially attached between two duplicated nodes that share many common neighbors. The NHD model can successfully reproduce various features of the yeast PIN that cannot be explained by the HD model.

## Results

**Figure 1:**
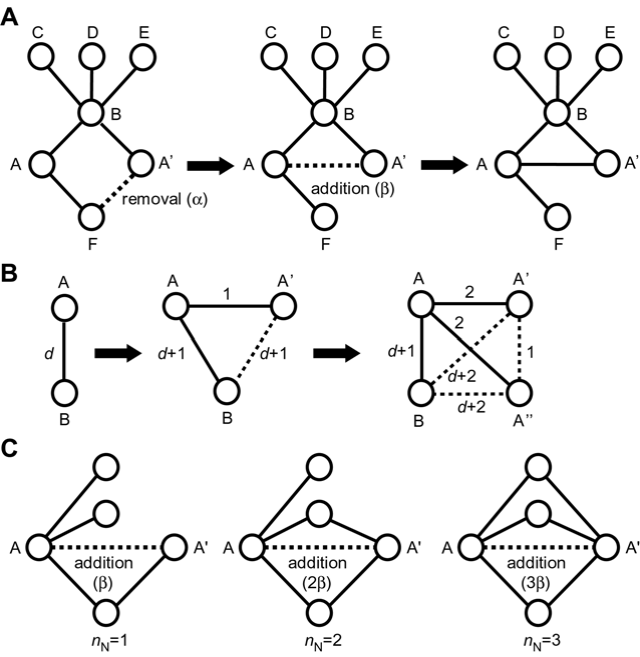
Simulation. (A) HD model. Node A is duplicated to generate node A′. Each of the links to node A′ is removed with a uniform probability *α* (left). Note that this method is based on completely asymmetric divergence [44], in which only one (A′) of the duplicated nodes is the target of removal of links. An HD link between node A and node A′ is attached with a uniform probability *β* (middle). (B) Evolutionary distance. When a node is duplicated, the evolutionary distance between each of the duplicated nodes and each of the other nodes in a network is assumed to increase by one due to mutations occurring in the duplicated nodes during the divergence process. Suppose that the evolutionary distance between node A and node B is *d* (left). After the duplication of node A to generate node A′ and the divergence of them, the evolutionary distance between nodes A and B, and that between nodes A′ and B become *d*+1 whether a link between nodes A and B and that between A′ and B are present or not (middle). (A dashed line indicates absence of a link.) The evolutionary distance between nodes A and A′ is defined to be 1 regardless of the presence of a link between them. After that, if node A′ is duplicated to create node A”, the evolutionary distance between nodes A and B continues to be *d*+1, while the evolutionary distances between nodes A and A′, A and A″, B and A′, and B and A″ become 2, 2, *d*+2, and *d*+2, respectively (right). (C) NHD model. In this model, the probability that a link is added between A and A′ is proportional to the number (*n*_N_) of common neighbors shared by these nodes.

In this study, we examined two models, the heterodimerization (HD) model and the non-uniform heterodimerization (NHD) model (see Materials and Methods for details). In the HD model, there are two parameters, the probability that a link is removed from one of the duplicated nodes (*α*) and the probability that a new link is attached between two duplicated nodes (*β*) (Figure 1A), which represents the probability that a duplicated protein is self-interacting and the interaction between two duplicated proteins survives after the divergence process. These parameters were determined to let <*k*> and <*C*> in a generated network be the same as those in the yeast PIN. To compare the number of HD links in a generated network with that in the yeast PIN, we defined an evolutionary distance (Figure 1B). Two nodes are defined to be homologous when the evolutionary distance between these nodes is lower than or equal to a given threshold value *d*_T_. The statistics of the networks generated by the HD model are shown in Table 1. The number of homologous pairs in the yeast PIN (*n*_H_ = 6,544) is between *n*_H_ for *d*_T = _3 (5,309) and that for *d*_T = _4 (8,337). However, the number of interactions between homologous nodes (*n*_HI_ = 395 and 514 for *d*_T = _3 and 4, respectively) is much larger than that in the yeast PIN (175). This observation is consistent with the investigation of the fly PIN by Ispolatov et al. [29], in which it was reported that the HD model requires a much larger number of HD links (270) than the actual number in the fly PIN (142) [32] to generate the 1,405 triangles present in the fly PIN.

**Table 1:** Statistics of the networks by the HD and NHD models and the yeast PIN

Model	*d*_T_	*α* a	*β* a	*n*_H_ b	*n*_HI_ c	*n*_HI_ */n*_H_	<*k*>^d^	*<C>* e	*<L>* f
HD model	1	0.725	0.061	1,312 (11)	140 (12)	0.107 (0.009)	3.73 (0.09)	0.066 (0.006)	6.45 (0.14)
	2	−	−	3,031 (27)	269 (19)	0.089 (0.006)	−	−	−
	3	−	−	5,309 (43)	395 (25)	0.074 (0.005)	−	−	−
	4	−	−	8,337 (65)	514 (31)	0.062 (0.004)	−	−	−
	5	−	−	12,363 (92)	628 (42)	0.051 (0.003)	−	−	−
NHD model	1	0.745	0.028	1,308 (11)	52 (6)	0.040 (0.005)	3.74 (0.07)	0.066 (0.006)	6.23 (0.12)
	2	−	−	3,030 (22)	105 (11)	0.035 (0.004)	−	−	−
	3	−	−	5,315 (42)	157 (17)	0.029 (0.003)	−	−	−
	4	−	−	8,351 (61)	208 (21)	0.025 (0.003)	−	−	−
	5	−	−	12,373 (86)	259 (28)	0.021 (0.002)	−	−	−
Yeast PIN^g^				6,544	175	0.027	3.74	0.066	4.85
Random^h^							3.74	0.00096	6.27

The number in parentheses represents the standard deviation calculated from 100 networks generated by simulations. −, the same as above.

a. Parameters used in the simulations. See Materials and Methods.

b. The number of homologous pairs. Two nodes are defined to be homologous when the evolutionary distance between the two nodes is *d*_T_ or less.

c. The number of interactions between homologous proteins.

d. The average degree.

e. The average cluster coefficient.

f. The average shortest path length.

g. The yeast PIN without self-interactions.

h. A random network that has the same <*k*> and *N* as those in the yeast PIN, where *N* is the number of nodes (3,891) in the yeast PIN. The values of <*C*> and <*L*> were calculated using the formulae <*K*>/*N* and log*N*/log<*k*>, respectively.

As was mentioned in the Introduction, the HD model can generate a network with a high <*C*>, because an HD link produces triangles. When two duplicated nodes share *n*_N_ common neighbors, *n*_N_ new triangles are created by an HD link between them (Figure 1C). Therefore, if a new link is attached between duplicated nodes more preferentially when a larger number of neighbors are shared between them, it is expected that HD links fewer than those required by the HD model can reproduce the high <*C*> in the PIN. For this reason, we examined the NHD model, in which the probability that a new link is added between duplicated nodes is proportional to the number of neighbors shared by these nodes. The probability of removing a link (*α*) and the proportionality constant to add a new link (*β*) were adjusted to let <*k*> and <*C*> in a generated network be the same as those in the yeast PIN. The results of simulations by the NHD model are shown in Table 1. Both *n*_H_ (6,544) and *n*_HI_ (175) in the yeast PIN are between the values for the NHD model with *d*_T_ = 3 and 4 (*n*_H_ = 5,315 and 8,351, respectively, and *n*_HI = _157 and 208, respectively). Moreover, the values of *n*_HI_/*n*_H_ for the NHD model with *d*_T_ = 3 and 4 are very close to that in the yeast PIN. Therefore, both a high <*C*> and a low *n*_HI_ were well reproduced by the NHD model. Table 1 also shows that <*L*> in both HD and NHD networks are similar to that in a random network, indicating that they are small-world networks. However, interestingly, <*L*> in the yeast PIN is much lower than that in a random network (see Discussion).

**Figure 2:**
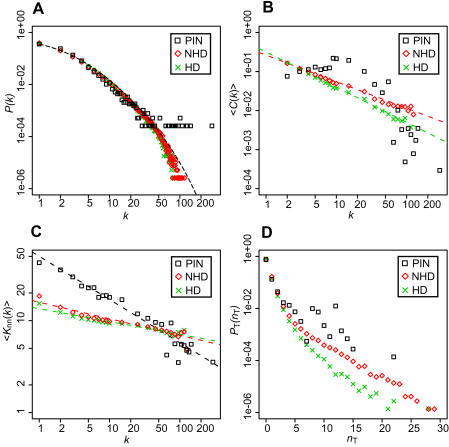
Properties in the networks by the HD and NHD models. Black squares, red diamonds, and green crosses show the values for the yeast PIN, the network generated by the NHD model, and the network by the HD model, respectively. The results for the HD and NHD models were obtained by taking the average among 100 networks generated by simulations. (A) Degree distribution *P*(*k*). The dashed line represents (*k*_0_+*k*)^−*γ*^e^−*k*/*kc*^ with *γ* = 2.7, *k*_0_ *= *3.4, and *k*_c_ = 50. (B) Distribution of the average cluster coefficient <*C*(*k*)>. Dashed lines in red and green indicate *k* ^−0.68^ and *k* ^−0.90^, respectively. (C) Distribution of <*K*_nn_(*k*)> indicating a disassortative structure. Dashed lines in black, red, and green represent *k* ^−0.47^, *k* ^−0.18^, and *k* ^−0.14^, respectively. (D) Distribution of *P*_T_(*n*_T_), the probability that a given link is contained in *n*_T_ triangles.

Figure 2A shows the degree distribution of the networks generated by the HD and NHD models and that of the yeast PIN. Although there is a discrepancy between the model networks and the yeast PIN for a large *k* (see Discussion), the results showed that both models can reproduce the degree distribution of the yeast PIN that follows a power law with an exponential cut-off. Figure 2B shows that <*C*(*k*)> in the networks generated by the models follows a power law, indicating that these networks exhibit a hierarchical structure. In the case of the yeast PIN, however, <*C*(*k*)> decreases following a power law as *k* increases only for a non-small *k* (*k*>10). This relationship was also observed in the previous studies [18], [19]. As shown in Figure 2C, both the HD and NHD networks display a disassortative structure <*K*_nn_(*k*)>∼*k^−ν^*, but the values of *ν* are smaller than that in the yeast PIN (see Discussion).

Figure 2D shows the probability *P*_T_(*n*_T_) that a given link is contained in *n*_T_ triangles in a network. For example, *n*_T_ = 2 for the link between nodes A and A′ (dashed line) in the middle of Figure 1C. The probability distribution *P*_T_(*n*_T_) is a statistic describing a spatial distribution of triangles in a network. In a network generated by the HD model, the spatial distribution of triangles can be regarded to be random, because addition of a new HD link occurs randomly. As shown in this figure, the distribution of *P*_T_(*n*_T_) in the yeast PIN is quite different from that in the network by the HD model, suggesting that the spatial distribution of triangles in the yeast PIN is highly skewed. In other words, in the yeast PIN, the extent of overlapping of triangles is larger than the expectation from a random distribution. On the other hand, the *P*_T_(*n*_T_) distribution for the NHD model is close to that in the yeast PIN. Therefore, the structure of a network generated by the NHD model is more similar to the PIN than that by the HD model.

In the HD and NHD models, self-interactions were not explicitly considered, though it was assumed that an HD link is created only when a self-interacting protein is duplicated. However, in the yeast PIN, the fraction of self-interacting proteins is only 0.049, and <*k*> increases slightly (3.84) when self-interactions are considered. The effect of self-interactions to other statistical properties of the yeast PIN is negligible (Figure S1). Therefore, it is expected that explicit consideration of self-interactions in a model does not essentially alter the results described above. We should also note that the fraction (0.049) of self-interactions in the yeast PIN is consistent with the value of *β* (0.028) in the NHD model. The fraction in the yeast PIN is much smaller than that (0.18) in the human transcription factor network, in which the statistical properties are considerably different between the networks with and without self-interactions [33].

We also examined the effect of gene deletions that are caused by mutations. For this purpose, we modified the NHD model by adding the process of random elimination of nodes (NHD+E model). However, the elimination of nodes did not essentially change the results (Table S1 and Figure S2).

## Discussion

In this study, we showed that the NHD model can successfully reproduce both a high <*C*> and a low *n*_HI_ in the yeast PIN, whereas the HD model cannot regenerate the value of *n*_HI_. We also demonstrated that the distribution of triangles in the yeast PIN is highly skewed and the skewed distribution can be reproduced by the NHD model but not by the HD model. These results suggest that the NHD model would reflect the actual evolutionary mechanism of PINs.

**Figure 3:**
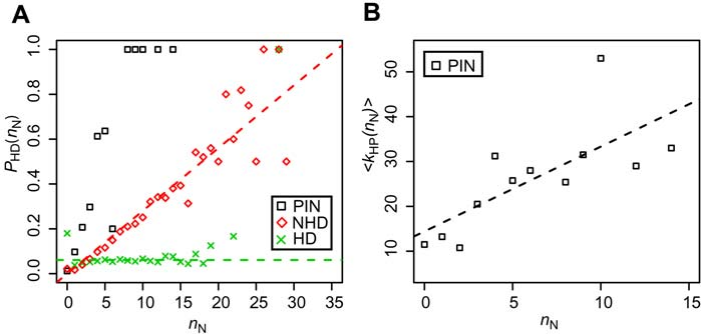
HD links in the yeast PIN and in the networks by simulations. Black squares, red diamonds, and green crosses show the values for the yeast PIN, the network generated by the NHD model, and the network by the HD model, respectively. (A) Distribution of *P*_HD_(*n*_N_), the probability that an HD link exists between two homologous proteins when they share *n*_N_ common neighbors (for *d*_T_ = 3). The slopes of the dashed lines are 0.028 (red) and 0 (green). The result for *d*_T_ = 4 is nearly identical to this result (data not shown). (B) Distribution of <*k*_HP_(*n*_N_)>, the average degree of proteins that are connected by HD links and share *n*_N_ common neighbors with their homologous proteins. The dashed line is a regression line (*r* = 0.73).

Is the NHD model biologically realistic? In the PIN evolution, when a self-interacting protein is duplicated, an HD link between duplicated proteins is added to the PIN. Some HD links survive in evolution, but other links disappear because of mutations occurring at interacting sites in one or both of the duplicated proteins. Therefore, in the HD model, an HD link is assumed to survive at a uniform rate. On the other hand, in the NHD model, it is assumed that the survival rate of an HD link is proportional to the number of common neighbors shared by the duplicated nodes. Figure 3A shows the probability *P*_HD_(*n*_N_) that two homologous nodes have an HD link when they share *n*_N_ common neighbors. This figure indicates that *P*_HD_(*n*_N_) is nearly constant regardless of *n*_N_ in the networks by the HD model. On the other hand, in the yeast PIN, *P*_HD_(*n*_N_) increases in proportion with *n*_N_, which is consistent with the NHD model. These observations suggest that, in the evolution of PINs, the survival rate of HD links is not uniform in terms of *n*_N_. Therefore, the NHD model appears to be realistic. The value of *P*_HD_(*n*_N_) in the NHD network is smaller than that in the yeast PIN for *n*_N_<15. This appears to happen because, in the NHD network, several protein pairs have very large values of *n*_N_, which is not the case in the yeast PIN. That there are no protein pairs with large *n*_N_ in the yeast PIN may be due to the high duplicability of low-degree nodes [34], which was not considered in the NHD model (see below).

Why, then, is the survival rate of HD links not uniform, but rather proportional to the number of common neighbors? One possible explanation is as follows. It has been reported that the degree of proteins in the yeast PIN is negatively correlated with their evolutionary rates [35]–[37], though this assertion is controversial [38]. Not surprisingly, proteins connected by an HD link that has a large *n*_N_ tend to have a high degree (Figure 3B). Therefore, the evolutionary rates of proteins in an HD link with a large *n*_N_ are expected to be low. If this is the case, the possibility of the occurrence of mutations at the binding sites would also be low, and thus the survival rates of HD links having a large *n*_N_ are thought to be higher than those of HD links having a small *n*_N_.

Although the degree distribution *P*(*k*) of the NHD network is generally in good agreement with that of the yeast PIN, the number of nodes with *k*>50 in the former is much smaller than that in the latter (Figure 2A). The average of the maximum degrees among the NHD networks is 75.2, while the maximum degree in the yeast PIN is 286. Moreover, though the NHD network exhibits a disassortative structure <*K*_nn_(*k*)>∼*k* ^−*ν*^, the value of *ν* is considerably smaller than that in the yeast PIN (Figure 2C). These discrepancies might be resolved by introducing a mechanism wherein low-degree nodes duplicate more frequently than high-degree nodes. Prachumwat and Li [34] reported a negative correlation between the degree of proteins and their duplicability. Due to the disassortative structure (Figure 2C), low-degree nodes have more links to high-degree nodes than to low-degree nodes. Therefore, as a result of frequent duplication of low-degree nodes, links between a high-degree node and a low-degree node are preferentially generated, and a high-degree node tends to gain new links. For this reason, with the mechanism of high duplicability of low-degree nodes, the degrees of hubs and the value of *ν* in Figure 2C are expected to become larger than the current values. Moreover, the lack of HD links with high *n*_N_ in the yeast PIN (Figure 3A) would also be explained with this mechanism, because HD links with high *n*_N_ should be rare if the duplicability of high-degree nodes is low.

Although PINs are generally considered to be small-world networks [4], [8], [10], [24], the <*L*> in the yeast PIN is much lower than that in a random network (Table 1). In fact, this observation is consistent with the previous result by Pastor-Satorras et al. [24], in which it was reported that <*L*> in the random network and that in their model network were 8.0 and 6.8, respectively. (However, they mentioned that these two values are “comparable”.) Therefore, the yeast PIN is an “ultra-small” network, in which <*L*> is lower than that in a random network. It is known that a scale-free random network is ultra-small [39], [40]. The yeast PIN can be randomized without changing the distribution of *P*(*k*) by using the random rewiring method [21]. In this method, two links in a network were chosen randomly, and these links were rewired by exchanging their connecting partners. After randomization of the yeast PIN, <*L*> (4.49) is similar, but <*C*> (0.010) becomes much lower than that in the yeast PIN. Therefore, the yeast PIN is far from a scale-free random network. Nevertheless, interestingly, the yeast PIN is an ultra-small network.

The difference in the value of <*L*> between the yeast PIN and the NHD network may be explained in the following way. It was reported that the removal of hubs drastically increases the value of <*L*> in the yeast PIN [41]. Therefore, the low <*L*> in the yeast PIN might be due to the fact that the number of hubs in the yeast PIN is larger than that in the NHD network (Figure 2A). (There are 17 nodes with *k*>50 in the yeast PIN, while the average number of nodes with *k*>50 among the NHD networks is 5.8.) In fact, if we eliminate all nodes with *k*>50 and all links connected to them from the yeast PIN and the NHD network, both <*L*> and <*C*> become similar between the two networks (<*L*> = 6.13 and 6.51, and <*C*> = 0.063 and 0.060 for the yeast PIN and the NHD network, respectively). It therefore appears that the presence of a large number of hubs in the yeast PIN would be the reason for a very low <*L*>.

The above discussion would indicate that the NHD model is merely a rough approximation of the actual mechanism of the PIN evolution. However, we should note that although our new model contains only two free parameters, it could well capture various aspects of the structure of the yeast PIN. The availability of high-quality interaction data from other species will thus help to clarify the architecture and evolution of PINs in greater detail.

## Materials and Methods

### Data

Human-curated interaction data of the yeast PIN were downloaded from the MIPS (Munich Information Center for Protein Sequences) database (http://mips.gsf.de) (18 May 2006) [3]. The interaction data are separated into several components that are not connected to each other; we used the largest component containing 3,891 proteins and 7,270 non-redundant interactions. Among these proteins, 191 proteins are self-interacting. The amino acid sequences of 6,736 yeast proteins were also obtained from the MIPS database. In order to estimate the number of interactions between homologous proteins in the yeast PIN, we identified homologous gene pairs. Self-against-self homologous searches were conducted for the 6,736 sequences by using the BLASTP program [42] with the cut-off E-value of 1e-5. We identified 6,544 homologous pairs (*n*_H_) and 175 interactions between these pairs of proteins (*n*_HI_) in the yeast PIN (see Table 1). The value of *n*_HI_/*n*_H_ did not essentially change when a more stringent cut-off E-value was used (*n*_HI_/*n*_H_ was 0.027 and 0.032 for the E-values of 1e-5 and 1e-10, respectively).

### Simulation

In this study, we used the “minimal genome” containing 113 proteins as the initial network, because the first living organism is assumed to have had at least 113 proteins [43]. We generated a single component random network containing 113 nodes with <*k*> = 3.74, which is the average degree of the yeast PIN. The evolutionary distance between two nodes present in the initial network was assumed to be infinity. We obtained very similar results when we started a simulation from the initial network containing only two nodes.

At each time step of simulation in the HD model, a new node is added to the network according to the following rules (Figure 1A). (1) A node is randomly selected (A) and is duplicated to generate a new node (A′), having the same interacting pattern as node A. (2) Each of the links to node A′ is removed with a probability *α* (completely asymmetric divergence [44]). (3) A link between node A and node A′ is created with a probability *β*. If node A′ does not have any links after these processes (all links to node A′ were removed and no links were created), node A′ is not added to the network. These processes were repeated until the number of nodes became 3,891, which is the number of nodes contained in the yeast PIN. In the NHD model, the probability that a new link is added between two duplicated nodes (A′ and A) is defined to be *βn*_N_ (when *βn*_N_≤1), where *n*_N_ is the number of common neighbors shared by these two nodes (Figure 1C). The probability is defined to be one when *βn*_N_>1. (However, there were no such cases in the simulations.) We performed simulations using various values of *α* and *β*. For a given *α* and *β*, we conducted simulations 100 times and computed the average of <*k*> and the average of <*C*> from the 100 networks. The values of *α* and *β* that could reproduce <*k*> (3.74) and <*C*> (0.066) in the yeast PIN were used (Table 1).

In the NHD+E model, the following process was added after the addition of new links at each step of the NHD model. A node in a network is randomly selected, and the selected node is eliminated from the network with a probability *δ* together with all interactions connecting to the selected node. If the selected node is connected to one-degree nodes, all of these one-degree nodes are also removed. We changed the value *δ* from 0.001 to 0.1 (see Table S1). The values of *α* and *β* were determined in the same way as in the NHD model.

## Supporting Information

Table S1 (0.04 MB DOC)

Figure S1 Properties in the yeast PIN with and without self-interactions. Red triangles and black squares show the values for the yeast PINs with and without self-interactions, respectively. (A) Degree distribution *P*(*k*). (B) Distribution of the average cluster coefficient <*C*(*k*)>. (C) Distribution of <*K*_nn_(*k*)>. (D) Distribution of *P*_T_(*n*_T_).
(0.78 MB TIF)

Figure S2 Properties in the networks by the NHD and NHD+E models. Black squares, red diamonds, and blue crosses show the values for the yeast PIN, the network generated by the NHD model, and the network by the NHD+E model with *δ* = 0.1, respectively. The results for the NHD and NHD+E models were obtained by taking the average among 100 networks generated by simulations. (A) Degree distribution *P*(*k*). The dashed line represents (*k*_0_+*k*)^−*γ*^e^−*k*/*k*_c_^ with *γ* = 2.7, *k*_0_ = 3.4, and *k*_c_ = 50. (B) Distribution of the average cluster coefficient <*C*(*k*)>. Dashed line in red indicates *k* ^−0.68^. (C) Distribution of <*K*_nn_(*k*)>. Dashed lines in black and red represent *k* ^−0.47^ and *k* ^−0.18^, respectively. (D) Distribution of *P*_T_(*n*_T_).
(0.82 MB TIF)
