# Supplementary material for: Non-Uniform Survival Rate of Heterodimerization Links in the Evolution of the Yeast Protein-Protein Interaction Network
Source: PLoS One. 2008 Feb 27;3(2):e1667. doi: 10.1371/journal.pone.0001667 (PMC2253498; doi:10.1371/journal.pone.0001667)
Supplement: Table S1 — (0.04 MB DOC) [file pone.0001667.s001.doc]

**Table S1. Statistics of the networks by the NHD and NHD+E models**

| Model | *δ*^a^ | *d*_T_ | *α*^b^ | *β*^b^ | *n*_H_^b^ | *n*_HI_^b^ | *n*_HI_*/n*_H_ | <*k*>^b^ | *<C>*^b^ | *<L>*^b^ |
| --- | --- | --- | --- | --- | --- | --- | --- | --- | --- | --- |
| NHD | 0 | 3 | 0.745 | 0.028 | 5,315 (42) | 157 (17) | 0.029 (0.003) | 3.74 (0.07) | 0.066 (0.006) | 6.23 (0.12) |
|  | − | 4 | − | − | 8,351 (61) | 208 (21) | 0.025 (0.003) | − | − | − |
| NHD+E | 0.001 | 3 | 0.745 | 0.028 | 5,309 (40) | 157 (16) | 0.029 (0.003) | 3.73 (0.09) | 0.066 (0.006) | 6.23 (0.13) |
|  | − | 4 | − | − | 8,336 (72) | 208 (20) | 0.025 (0.002) | − | − | − |
|  | 0.01 | 3 | 0.74 | 0.028 | 5,266 (39) | 159 (15) | 0.030 (0.003) | 3.73 (0.08) | 0.066 (0.006) | 6.31 (0.14) |
|  | − | 4 | − | − | 8,253 (71) | 211 (19) | 0.026 (0.002) | − | − | − |
|  | 0.1 | 3 | 0.695 | 0.026 | 4,862 (48) | 158 (16) | 0.032 (0.003) | 3.76 (0.10) | 0.068 (0.007) | 7.03 (0.39) |
|  | − | 4 | − | − | 7,446 (71) | 210 (19) | 0.028 (0.003) | − | − | − |
| Yeast PIN^b^ |  |  |  |  | 6,544 | 175 | 0.027 | 3.74 | 0.066 | 4.85 |

The number in parentheses represents the standard deviation calculated from 100 networks generated by simulations. −, same as above.

a. A randomly selected node was removed with a probability *δ* in each step. See Materials and Methods.

b. See Table 1.
