# Supplementary figures and images for: Non-Uniform Survival Rate of Heterodimerization Links in the Evolution of the Yeast Protein-Protein Interaction Network
Source: PLoS One. 2008 Feb 27;3(2):e1667. doi: 10.1371/journal.pone.0001667 (PMC2253498; doi:10.1371/journal.pone.0001667)

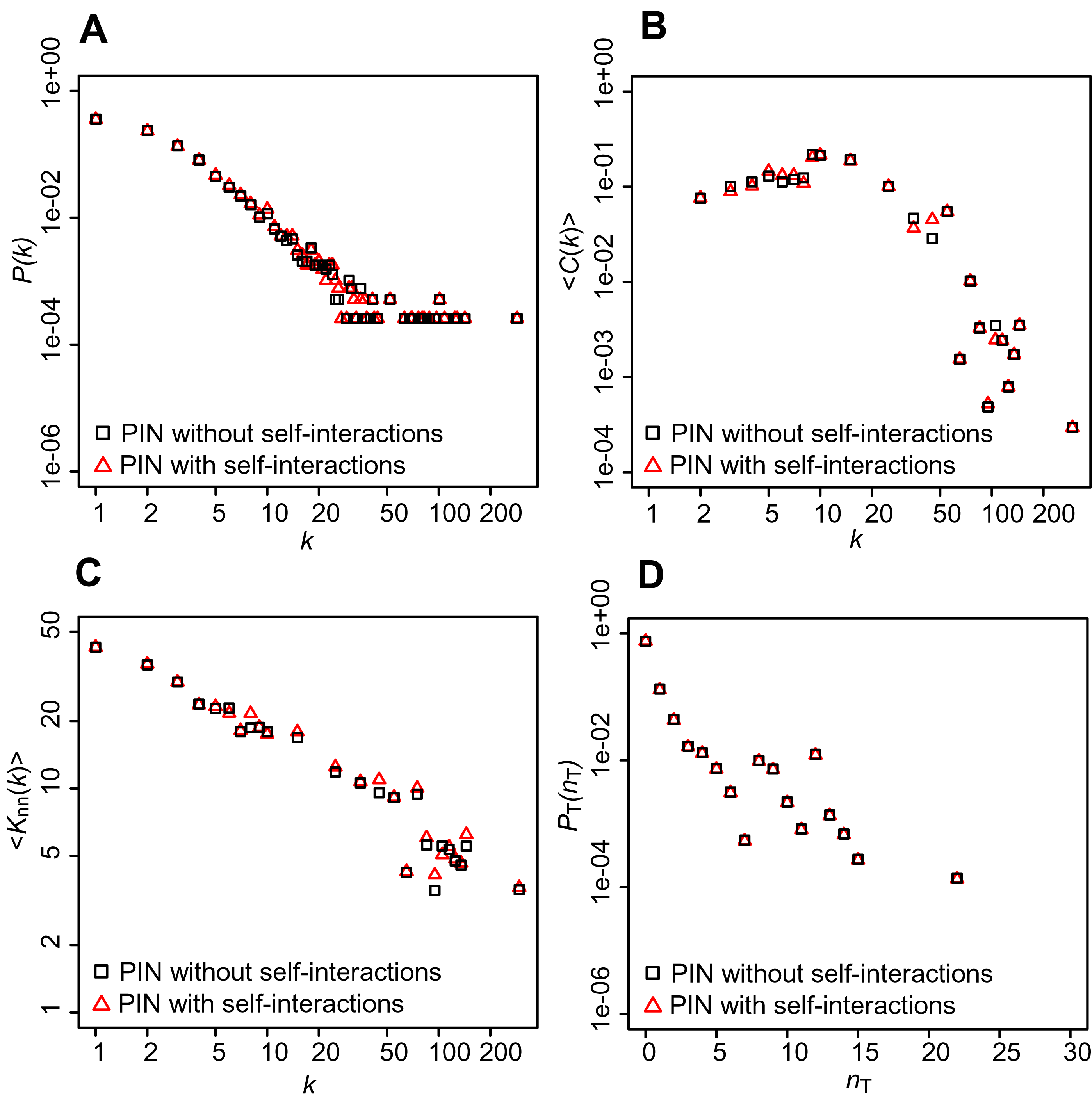

Supplement: Figure S1 — Properties in the yeast PIN with and without self-interactions. Red triangles and black squares show the values for the yeast PINs with and without self-interactions, respectively. (A) Degree distribution P(k). (B) Distribution of the average cluster coefficient <C(k)>. (C) Distribution of . (D) Distribution of P T(n T). (0.78 MB TIF) [file pone.0001667.s002.tif]

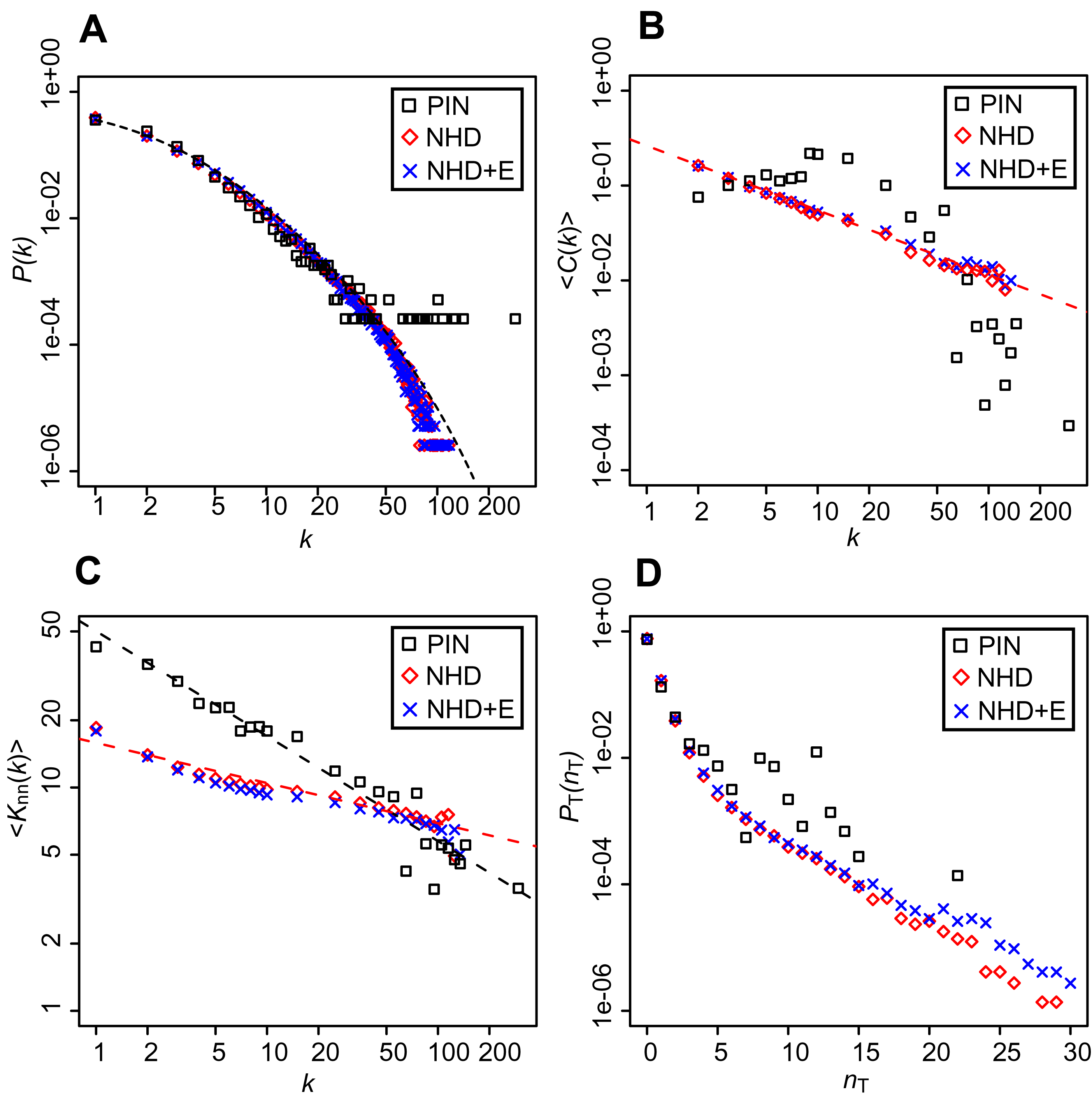

Supplement: Figure S2 — Properties in the networks by the NHD and NHD+E models. Black squares, red diamonds, and blue crosses show the values for the yeast PIN, the network generated by the NHD model, and the network by the NHD+E model with δ = 0.1, respectively. The results for the NHD and NHD+E models were obtained by taking the average among 100 networks generated by simulations. (A) Degree distribution P(k). The dashed line represents (k 0+k)−γe−k/kc with γ = 2.7, k 0 = 3.4, and k c = 50. (B) Distribution of the average cluster coefficient <C(k)>. Dashed line in red indicates k −0.68. (C) Distribution of . Dashed lines in black and red represent k −0.47 and k −0.18, respectively. (D) Distribution of P T(n T). (0.82 MB TIF) [file pone.0001667.s003.tif]
